# An Active Learning Activity to Reinforce the Design Components of the Corticosteroids

**DOI:** 10.3390/pharmacy6010016

**Published:** 2018-02-05

**Authors:** Stephen R. Slauson, Prashant Mandela

**Affiliations:** 1 School of Pharmacy, University of Saint Joseph, 229 Trumbull Street, Hartford, CT 06103, USA; 2 School of Pharmacy, Notre Dame of Maryland University, 4701 North Charles Street, Baltimore, MD 21210, USA; pmandela@ndm.edu

**Keywords:** active-learning, medicinal chemistry, large lectures, group learning, corticosteroids

## Abstract

Despite the popularity of active learning applications over the past few decades, few activities have been reported for the field of medicinal chemistry. The purpose of this study is to report a new active learning activity, describe participant contributions, and examine participant performance on the assessment questions mapped to the objective covered by the activity. In this particular activity, students are asked to design two novel corticosteroids as a group (6–8 students per group) based on the design characteristics of marketed corticosteroids covered in lecture coupled with their pharmaceutics knowledge from the previous semester and then defend their design to the class through an interactive presentation model. Although class performance on the objective mapped to this material on the assessment did not reach statistical significance, use of this activity has allowed fruitful discussion of misunderstood concepts and facilitated multiple changes to the lecture presentation. As pharmacy schools continue to emphasize alternative learning pedagogies, publication of previously implemented activities demonstrating their use will help others apply similar methodologies.

## 1. Introduction

The notion that actively engaging students versus passively lecturing increases long-term learning and critical understanding of material has been postulated as early as the 1960’s [1]. As educational associations began studying and recommending alternative teaching pedagogies, the opportunities to engage students in active learning concepts grew exponentially [2]. Although these options lead to positive outcomes resulting in better student performance in many cases, more discipline specific examples coupled with performance outcomes need to be reported in the literature to facilitate quicker and easier adoption by overburdened faculty members who already have well-reviewed lectures ready for use.

Medicinal chemistry is a very interesting area to apply active learning activities due to the variance of chemical knowledge in traditional pharmacy students. The challenge of lecturing to such a heterogeneous class is balancing coverage of important clinical concepts without incorporating extensive chemical rationalization. To compound this issue, the course is taught by chemists who intuitively associate these concepts with high level chemical understanding and very little direction on methods to simplify the information or time to teach the basic chemical concepts that some students lack. Due to this combination of factors, many student pharmacists appear to memorize their way through the chemistry portions of the curriculum with very little understanding of why they are taught. It is absolutely terrifying that we are educating a generation of pharmacists who may not be equipped to make the leap between the chemical structure of a pharmaceutical agent and its activity or physical characteristics By implementing active learning strategies, instructors can require students to focus critical evaluation of drug structure and/or physical characteristics to pharmacological or therapeutic situations while answering individual student questions one-on-one through monitoring. This model also affords students with superior chemical knowledge the opportunity to help less equipped students through individual interaction. Meanwhile, lecture activities can be constrained to the clinically correlated aspects of the drug class. 

Analysis of the existing literature with regards to active learning activities in chemistry related to the education of pharmacists demonstrates a relative lack of publications in this area. While a quick literature search of active learning results in greater than 35,000 hits, this field is quickly narrowed to 73 with the term medicinal chemistry. Individual analysis of these hits shows 49 to be orthogonal to this study (learning from activity cliffs, active machine learning, and active components of various natural extracts were the bulk of these 49). The other 24 are divided into abstracts of papers from national or regional ACS meetings (8) and publications in the area of interest (16). Although abstracts are useful for monitoring particular research employed across a variety of disciplines or geographic areas, they fail to provide the in-depth information required to make large curricular changes. Of the sixteen papers reported, six emphasized curricular models or assessment models (not individual activities) [3,4,5,6,7,8], three incorporated strategies for lab-specific training [9,10,11], while the remaining articles focused on individual activities. Of these seven, two utilized modeling software to reinforce drug-target interactions [12,13], three question-writing activities [14,15,16], one a series of crossword puzzles [17], and the last a unique performance enhancing drug (PED) module [18]. Of the dearth of possible active-learning activities in medicinal chemistry, additional reports could certainly facilitate the adoption of robust activities demonstrating an improvement in student learning and retention.

## 2. Materials and Methods 

The University of Saint Joseph School of Pharmacy is a modified-block, mastery based curriculum model that utilizes novel teaching pedagogies in both an individual and a group setting. Active learning concepts such as turning point questions, think-pair-share techniques, and muddiest point/summary exercises are utilized regularly during classroom activities. The students are also divided into 12 collaborative learning groups that demonstrate a cross-section of the gender, cultural, personality profiles and academic capabilities contained within the class. These groups are revised after each semester to allow for different soft skillsets to be developed—such as learning to work as a team, working with difficult teammates, and leadership skills. Several learning models such as problem-based learning, flipped classroom, and active learning are utilized in the group setting. 

Our first year curriculum during the spring and summer semesters focuses on the integration of physiology, pathophysiology, pharmacology, and medicinal chemistry concepts in classes that cover distinct anatomical systems (i.e.,—central nervous system, cardiovascular system, endocrine system, etc.). The courses are further divided into segments usually dedicating 3 h towards the general coverage of a particular concept with any teaching, learning, formative assessments, and group activities included within this period. Each segment is associated with specific learning objectives and assessment questions targeting that segment are mapped to these objectives through Examsoft. This article is detailing an active learning module applied during the pharmacochemistry of the endocrine system course.

The endocrine course is taught over five days, allowing material to be distributed into 10 separate 3 h modules. During the years discussed by this article, our course was divided as shown in Table 1. The first segment of day 3 is a careful, yet thorough overview of the physiology, pathophysiology and pharmacology of the corticosteroids as they apply to disease states, possible targets for therapeutic intervention, and general guidelines governing therapeutic use in this area. The therapeutics section of this subject is taught the following year by practitioner colleagues; therefore, mention of therapeutics in this course is targeted at helping student comprehension rather than application to practice. For instance, students are instructed that glucocorticoids can be classified by potency and local/systemic distribution, with each parameter taken into account when devising patient care plans. Loose guidelines, such as short duration treatment of strong systemic glucocorticoids to prevent adverse effects, are relayed to help students understand that each class and formulation of drug has specific indications for which it is most suited. This facilitates higher-level discussion and bypasses comments such as “I would use X because it is the most potent”. 

The second segment of day 3 builds upon these concepts covering the actual drugs including names, structures, metabolism, duration, half-life, physical characteristics, potency enhancing functional groups, and modifications utilized for formulation. After a 45–55 min lecture, the class is released to their collaborative learning groups and assigned the activity included as Appendix A. During this session, groups must propose one glucocorticoid drug and one mineralocorticoid drug. As part of the design process, the group must focus their design on a therapeutic area (such as local inflammation or Addison’s disease) with the concomitant formulation and strength of the agent matching its use. This requires the students to apply the knowledge from the previous segment covering the physiology and pharmacology of the system, their pharmaceutics knowledge from the previous semester, and integrate it with the structure activity relationships they learn in this segment. The groups then must nominate one member to take part in the “FDA review panel” that will ask questions to the group as they present their proposals. The panel design is twofold: (1) it encourages questions to every group from the students themselves without making them feel like they are “bullying” their classmates (in fact, it creates almost a competition to see if they can find flaws in their colleagues’ designs) and (2) it often elucidates concepts that are not clear. The group design portion of the activity is typically limited to 45–50 min to allow adequate time for presentations. 

During the presentation aspect, each group presents their design and answers questions proposed by the panel or other classmates. This section typically runs between 75–90 min. To personalize and increase the creativity aspects of the assignment, groups are encouraged to give their drug a name. As instructors, we provide some amount of leeway to allow presentations to happen organically, but intervene on occasion to clear up misunderstood concepts or to keep things moving in a timely fashion (such as groups trying to delay their own presentation by asking trivial questions). After the activity, we provide positive feedback about the successful integration of concepts and reiterate corrections to misconceptions discovered during the activity. Any design aspects that are interesting yet unknown to the instructors are investigated after class and reviewed the first 5 min of the next segment.

This activity is designed to actively reinforce the structure activity relationships (SAR) of the corticosteroids. During the discussion of group proposals, concepts such as structural differences between an agonist and an antagonist, a mineralocorticoid and a glucocorticoid, and strong versus weak glucocorticoids are covered. The influence of 11β-hydroxy steroid dehydrogenase and the blocking effect of C9α-substituted drugs on this enzyme are thoroughly examined. Numerous other more specific trends are reviewed including the binding influence of C16/C17 substitutions, metabolism based modifications leading to prednisone and methylprednisolone, and the low systemic availability of metabolically labile groups.

This study was approved by the institutional review board of the University of Saint Joseph with the IRB protocol number 17-0073. 

## 3. Results and Discussion

For ease of discussion, the results of this study are divided into (1) the design aspects of the proposed agents and (2) the performance of the class on the related objectives mapped to this segment of material. 

### 3.1. Group Designs

During the 2013–2014 academic year, the course was taught in a non-integrated fashion with the physiology and pharmacology covered in seven segments and the medicinal chemistry and pharmacokinetic/pharmacodynamic principles in the remaining three (repeating the same format as the previous year). This approach was poorly reviewed by students (3.97/5.00) and integrated the following year. Although the course has undergone extensive changes during the last four years in regards to how and when each topic is covered, the corticosteroid discussion has been largely unchanged with regards to the lecture portion. Major changes since the 2014–2015 academic year include updating the content and altering the presentation of misunderstood concepts from previous years. The integration, updating, and three years of experience have resulted in better reviews for the course in 2016–2017 (4.44/5.00). As mentioned previously, the students were assigned to one of twelve collaborative learning groups of six to eight students. This course is the first of the summer semester and represents the first course with the students’ third assigned peer learning group. 

#### 3.1.1. Glucocorticoids

**Figure 1:**
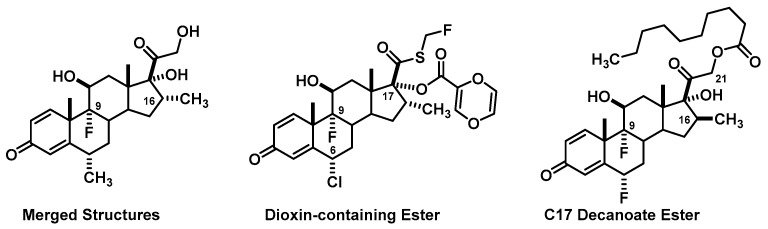
Common Glucocorticoid Design Elements.

For the design of the glucocorticoid drug, 42% of groups (16 of 38) merged structural components of the marketed drugs we discussed in the lecture session (Figure 1). Examples of these are different ester chains at the C21 hydroxy, combinations of a C6α-halogen and C9α-halogen, varying usage of a C17α /C16α/C16β substitution with either a methyl or hydroxy, and use of acetonides or esters at C16 and C17. In an effort to combine the binding interaction of budesonide’s acetonide with an increase in lipophilicity, several groups exchanged the C16 ester chain for some rather novel derivatives including one that had a 1,4-dioxin containing ester. Several others utilized different esters at C17 mostly utilizing ester chains seen in other steroids or from previous courses. Only 2 groups from this section had extensive questioning about their design, both claiming a topical agent with no lipophilic groups added. Although this is not necessarily a flaw, the intent is to draw upon their pharmaceutics knowledge and design drugs that are more lipophilic.

**Figure 2:**
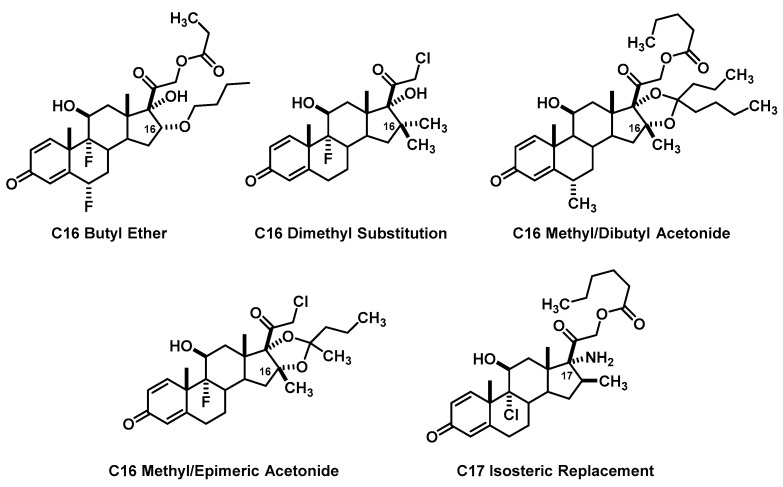
Main Groups of C16/C17 Oriented Changes.

The other 58% of groups can be organized into a few different specific categories of substitution (Figure 2). The first is a simple extension of the C16α methyl to a butyl group. Although no direct correlation to this exists in the literature, it is reasonable that if larger acetonides are facilitated, then the simple butyl substituted derivative would not be completely precluded from binding. Another similar design incorporated a butyl-ether, which is shown to have anti-inflammatory properties [19]. The second are the C16 di-substituted examples. From a student perspective, if either the C16α or C16β methyl afford higher potency for anti-inflammatory effects and protection from the unwanted mineralocorticoid effects, then having both at the same time would afford an even better drug. In fact, three patents from the 1960’s invoke this design aspect and report anti-inflammatory properties [20,21,22]. Three groups combined this structural element with those seen in the marketed drugs for their design. A fourth group in this category utilized a C16β methyl with a C16α ketone (methyl). The last of the examples utilized the C16β methyl with a C16α hydroxy incorporated as an acetonide with the C17α hydroxyl [23]. They also utilized a di-butyl acetonide similar to budesonide’s epimeric version to assimilate more lipophilicity in their design. Another group had a similar design also incorporating a C16/C17 acetonide, but with methyl/butyl chains. These larger acetonides are also confirmed to act as glucocorticoids in the patent literature [24]. The third substitution pattern is isosteric replacement, covered in our introductory medicinal chemistry course some 6 months prior focusing on a hydride displacement table. Some examples are simple replacement of the C16 or C17 hydroxy with an amine or replacement of the ester oxygen with an NH [25,26].

**Figure 3:**
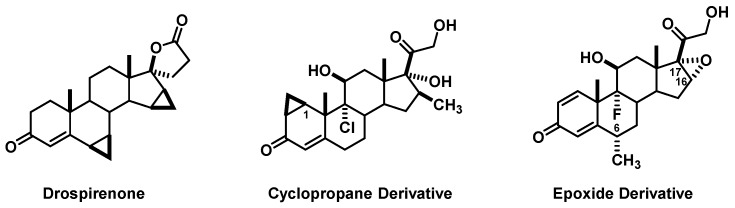
Novel Glucocorticoid Designs.

There were also several less prevalent, but novel modifications adapted from their general chemical understanding (Figure 3). When discussing drugs such as drospirenone, the idea of utilizing cyclopropanes as a way to modify bond angles/lengths or introduce geometric isomerism is presented. As such, one group replaced the Δ1-alkene present in many glucocorticoids with a cyclopropane. An additional unusual suggestion included a C16/C17 α-epoxide instead of an acetonide, though we teach that this acetonide is responsible for binding interactions as evidenced by budesonide. Another group argued that extension to a C6-hexane chain would accentuate the binding increase seen with C6-methyl derivatives in addition to increasing lipophilicity.

In the glucocorticoid molecules, very few design elements would be classified as mistakes—most often ill-advised due to evidence that they would be counterintuitive to their proposed drug. Examples of mistakes include absence of the C17β chain (or any substitution at C17), removal of multiple elements such as the C11 oxygen coupled with another pharmacophoric element, and moving pharmacophoric elements to different positions (such as the C17 chain to C16 and proposing it would have better potency and similar selectivity). Due to the conserved nature of steroids and the inherent challenge of building selectivity, the students are advised that the above examples would be a very risky use of their company’s resources.

**Figure 4:**
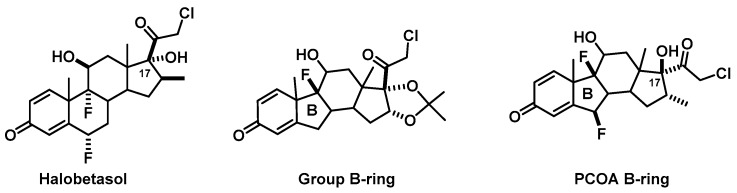
5-Membered B-Ring Corticosteroids and Halobetasol.

One noteworthy proposal was a five-membered B-ring structure for one group’s glucocorticoid. This group was part of the 2013–2014 class and their presentation ignited significant discussion from the panel and class about the change of the basic steroid pharmacophore—interesting considering some of the changes proposed by other groups as discussed above. Confident that the structural change was reported somewhere in the literature, we instructed the students to move forward and researched the proposal after class. The next morning, our findings were discussed and the class informed that this was a very logical attempt that was reported, but unfortunately resulted in lower affinity compounds with less selectivity [27]. This is mentioned because the following academic year (2014–2015), this same cadre of students were the first from our school to take the Pharmacy Curriculum Outcomes Assessment (PCOA). The NABP’s (National Association of Boards of Pharmacy) example question for the ordered response format involves the rank ordering by potency of topical corticosteroids with one structure having a five-membered B-ring (Figure 4). The substitution pattern is basically halobetasol (one of the most potent topical glucocorticoids) with the B-ring exchanged. This particular structure has other issues in regards to typical steroid structure such as the inverted stereochemistry of the halogens and the C17 position, but the prominent piece is the B-ring change due to the conformational effects. The coverage of alternative pharmacophoric ring systems as corticosteroids is not a concept covered in traditional medicinal chemistry courses, and certainly not one that would have been covered in this course had it not been for this group’s design. In fact, this observation is evidence that affording students creative license during active learning exercises can lead to higher order critical thinking and extended learning for both the students and the professors.

#### 3.1.2. Mineralocorticoids

**Figure 5:**
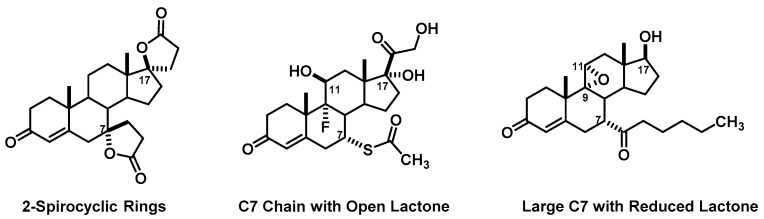
Mineralocorticoid Antagonist Designs.

In contrast to the glucocorticoids where all groups designed agonists, 21% (8 of 39) of groups designed antagonists for the mineralocorticoids with one group designing both an agonist and an antagonist. Of the antagonist designs, the 4 groups that kept the spirocyclic ring at C17 made other structural changes, mostly combining substitution patterns or making small changes to the structures of eplerenone and spironolactone, such as exchanging the C7 chain from one to the other or extending this chain with more carbons. One very interesting design incorporated a second spirocyclic ring at the C7 position (Figure 5). Aside from the probable difficulties of synthesizing such a derivative, this is the critical thinking aspect we are trying to encourage. If the β-ketol of aldosterone can be switched to a reverse lactam (17β-20 carbonyl versus 17β-oxygen) and still retain selectivity for the mineralocorticoid receptor, why couldn’t the thioester at C7 be switched for a spirocyclic lactone? Literature searches for similar structures do not reveal any attempts in the mineralocorticoid field, but do reveal two C7- disubstituted cholesterol derivatives and one report of C7-disubstituted pregnanes and androstanes, at least insinuating that similar designs have been investigated [28,29,30]. 

The antagonist contributions were split between keeping the spirocyclic ring at C17 with 50% (4 of 8 groups) opting to either open it to the β-ketol, change it to a ketone, reduce it to a hydroxy (C17β-hydroxy), or remove the C17-substitution altogether (leaving a CH_2_ at C17). The β-ketol group incorporated a C9α-fluoro and a C7 substituiton mimicing spironolactone to argue that they would have both mineralocorticoid receptor selectivity and antagonist effects (Figure 5). The group that exchanged the spirocyclic ring for a methyl ketone incorporated a C9α-fluoro to block 11-β HSD, but left off the C11β-hydroxy demonstrating an incomplete understanding of the concept. The group that reduced the C17 to a hydroxy used a C9–C11 epoxide for selectivity and a 6-carbon ketone chain at C7 to ensure antagonistic effects (Figure 5). The group that removed the C17 ring incorporated a C7 methyl ketone and an episulfide in place of the typical epoxide from C9–C11 indicating knowledge of isosteric replacement. A quick literature search of this structure returns no hits, but analysis based on knowledge of steroid structure would predict this design to be premiscuous considering the conserved nature of substitutions at C17.

**Figure 6:**
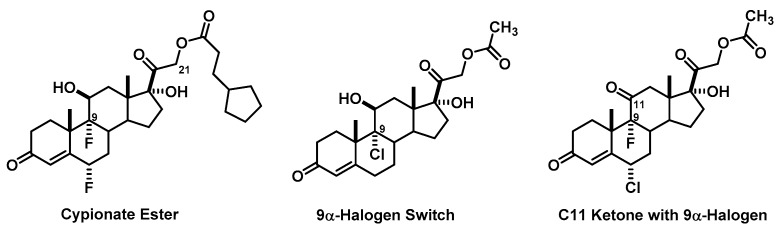
Common Mineralocorticoid Agonist Designs.

Of the 31 groups that proposed mineralocorticoid agonists, 52% (16 of 31) utilized a mixture of structure activity relationship components and lipophilic substitutions such as cypionate esters to design either oral or injectable depot formulations combining a C9α-fluoro or C9α-chloro with an unsubstituted C16 (Figure 6). These groups confirmed adequate understanding of design aspects of the corticosteroids and applied them successfully. The other 48% can be divided in groups with structural flaws (39%) and those with unique design aspects (10%). The structural flaws consisted of removing the C11β-hydroxy, yet employing a C9α-halogen to block 11β-HSD (5 groups) or removing C17 substitution completely (4 groups). One group used a C9α-bromo to block 11β-HSD: Although extensive application knowledge of undergraduate organic is not expected of our students, this is classified as a design flaw and was noted during the presentation without professor involvement. The other two groups in this category completely missed the main objective behind the 11β-HSD discussion, designing molecules with either a C11 ketone coupled with a C9α-fluoro or a C11β-hydroxy with no C9α-substitution. In both instances, the “FDA panel” immediately questioned those design aspects and corrected the flaw. 

**Figure 7:**
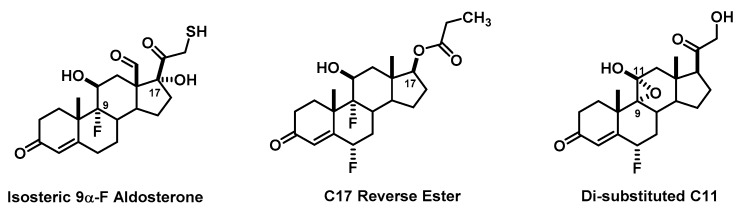
Advanced Mineralocorticoid Agonist Designs.

The remaining three groups involved more critical analysis in their design (Figure 7). One group simply converted the C17β-ketol to the thio version (OH to SH) again relying on their isosteric knowledge from a course delivered some 6 months prior. Drawing on their chemistry knowledge of thiols, they even commented that it may smell—an excellent observation for students at this level of chemical knowledge. Upon further investigation, many mercapto-ketones (such as 3-mecapto-2-butanone or 1-mercapto-2-propanone) are actually used in the fragrance and flavor industry suggesting that this might not be an issue with their design [31]. Another group incorporated a reverse ester at C17 drawing upon the lactone orientation in the mineralocorticoid antagonists eplerenone and spironolactone. Literature analysis supports our intuition that these would most likely have androgenic effects, despite the incorporation of the C11β-hydroxy [32]. The last group incorporated both a C11β-hydroxy and an alpha oriented C9–C11 epoxide. Their rationale was that since the epoxide seemed to provide more selectivity to eplerenone over that observed in spironolactone, then it could provide the same advantage here. This again falls into a category that appears very difficult to synthesize, but it is important to remember that these are pharmacy students (not medicinal or organic chemists) and the design rationale exhibits application of concepts from different pharmacological categories. 

#### 3.1.3. Therapeutic Indications

In addition to two structures, students are also asked to provide usage information relevant to their design. Most groups (68%) simply note the route of administration (oral, topical, or injection) either in written form or verbally when asked by the panel; however, some groups (32%) designate a disease state, dosing, expected adverse effects, and precautions. Keeping in mind that these students are one year removed from undergraduate coursework, this extra information can have both favorable and unfavorable consequences. A majority of the time (75%—9 of 12 groups), this extra information is correct and results in fewer questions from the panel and audience.

Occasionally, however, a group incorrectly applies a newly learned concept and is questioned by their peers. Two such occasions presented here are one group applying a mineralocorticoid agonist for Cushing’s syndrome (although mineralocorticoid antagonists are used for Cushing’s, this was clearly a confusion between Cushing’s and Addison’s disease) and another using a mineralocorticoid for arthritis and asthma (again just a confusion between the uses of gluco versus mineralocorticoids). The third occasion was the use of the 6β-hydroxy prednisolone active metabolite as a drug. The group proposed the use of this metabolite to circumvent metabolism issues associated with concomitant administration of phenobarbital (a known inducer of many CYP450 enzymes). This is most likely a conclusion drawn from our focus on drug-drug interactions caused through CYP induction/inhibition discussed in the central nervous system class. We especially focus on CYP2D6 polymorphism and how active metabolites can theoretically be used to reduce adverse effects. Although the students thought process was not completely clear in this particular case, they were the only group in this study to choose an active metabolite—a product life-cycle management strategy discussed in previous courses. Taking into account that the previous segment of material was the first in-depth discussion of Addison’s and Cushing’s disease for most students and the fact that detailed therapeutics was not discussed in this course, these cases represent a very small contingent and afford the opportunity to reinforce the difference between these two disease states in a different learning environment.

#### 3.1.4. Drug Names

Many groups (55%—21 of 38) choose to name their new drug candidates as well. This aspect does not represent any part of the focused objective, but does introduce some humor into the presentations. It also helps some of the less clinically oriented students to recognize the aspects of both the chemical and trade names of marketed drugs. Lastly, groups incorporating names for their drugs represent the groups with the most creative designs. 16 of the 21 groups (76%) with names contained elements associated with more critical thinking and 4 of the remaining 5 (80%) provided simplistic chemical names based on the original drug modified. This suggests a correlation between groups using more creativity in design elements and those extending this creativity into naming.

Of the 21 groups providing a name, 7 groups (33%) used variations of the group name or an individual group member’s name, one of the favorites being Dr. X’s Depot for a design containing a decanoate ester on the C21 hydroxy. An additional 2 groups (10%) used some aspect of the state (Connecticut) in their name, such as Connecticutsone for a C21 fluorine ester derivative of prednisone. Five groups (24%) used direct derivatives of known drugs and matched chemical names to their designs. An example is the name “clodrocortisone” for the chlorine replacement of the fluorine in fludrocortisone. The remaining 7 groups (33%) provided trade names for their products (these groups also included therapeutic indications). Most of these were very descriptive; for instance “Flowxtreme” for a mineralocorticoid antagonist design drawing on its diuretic action or “Rheumacure” for a glucorticoid designed to combat rheumatoid arthritis.

### 3.2. Class Performance

In view of the fact there is one broad objective (C03) assigned to this segment of course material, we can easily relate students’ performance on this material by reviewing metrics for this objective on the assessment.

C03: Given a drug that acts as or inhibits the actions of adrenocortical hormones, discuss its chemistry, including structural determinants of action and receptor selectivity

The metric predicted in this study is either a consistent increase in performance on objective C03 over the years shown, or a dramatic increase in year 1 and consistent performance thereafter. When evaluating the data trends in Table 2, neither of these trends is immediately evident. Basically, the class performed below expectations in 2013–2014, much better in 2014–2015, then leveled to a low to mid-70’s average the next two years. In 2012–2013, a case study was utilized asking the user to determine the best drug for lung inflammation resulting from aerosolized poison ivy with choices of fludrocortisone, prednisolone, and clobetasol proprionate. This activity asked students to differentiate structurally between glucocorticoids and mineralocorticoids and apply their pharmaceutics or literature searching skills to find that clobetasol is typically topical. Replacing this case study with the drug design activity described above theoretically should have resulted in more significant application of the objectives and subsequently better recall during examination.

Comparison of student performance on this objective (C03) compared to performance on all objectives C (corticosteroids), chemistry questions, pharmacology questions, overall assessment, or percentage of re-assessors (Table 2) provided no additional trends or evidence to support the hypothesis. Performance on chemistry versus pharmacology was nearly equivalent (with the exception of 2014–2015) and the percentage of students re-assessing the exam similar. The class average increased marginally over the four year period, but was most likely a result of other variables.

In light of the general vagueness of the objective, performance can also be narrowed to 5 main aspects students are expected to master for the assessment—corresponding to the five assessment questions written for this topic (Table 3). The first concept is general structure-activity relationships of the glucocorticoids. Examples include the increased rate of metabolism of the carbothioate in fluticasone resulting in very low systemic absorption or the receptor interaction/potency increase of the furoate ester in mometasone. The second concept is metabolism, or more precisely the substitutions made to hydrocortisone and subsequent analogs to extend duration or increase potency based on analysis of metabolic pathways. The third concept is the ability of 11β-hydroxysteroid dehydrogenase (11β-HSD) to interconvert between the active (C11β-hydroxy) and inactive (C11-ketone) forms of cortisone/prednisone in the liver and inactivate the same in the kidney to circumvent dramatic mineralocorticoid effects. The fourth revolves around the understanding that the C9α-halogen substitution of corticosteroids increases the potency at the glucocorticoid receptor, but inhibits 11β-HSD thereby dramatically increasing the mineralocorticoid effects. As evidenced in marketed drugs, 16-position substitutions with a methyl or hydroxy interfere with mineralocorticoid activity and are therefore necessary when a C9α-halogen is present to counteract the mineralocorticoid effects and have a drug with predominantly glucocorticoid effects. The assessment question typically asks students to identify a drug with the most glucocorticoid versus mineralocorticoid effects in a field of 3–4 structures. The last concept is simply recognition of structural components of the mineralocorticoid agonists/antagonists. The data corresponding to these five topics over the last four years fails to show a general upward trend or a consistent performance level. After extensive analysis of question-to-question variance between these four exams, there are no differences in the format of the questions, language used, Bloom’s taxonomy, or apparent difficulty associated. The only explanations offered are that each cadre of students are different, ask different questions leading to emphasis on different aspects of the information, and perform slightly differently in any particular course or aspect of a course than previous cadres.

There are several confounding variables to be taken into account when analyzing the data provided. The first and largest factor is that two new faculty members taught the course for the first time in 2013–2014 versus two established faculty members in 2012–2013. The faculty members were new to teaching, new to this course, and new to a mastery-based curriculum utilizing group-format active-learning. The low pass-rate for individual questions in 2013–2014 and 2014–2015 might stem from inexperience writing mastery based multiple choice questions instead of failure of students to master the objective. Another variable for the data is that the course was taught in an integrated fashion starting in 2014–2015, with the 2013–2014 course offering discontinuous with three sessions of medicinal chemistry and seven sessions of pharmacology (the same format as in 2011–2012). The third major variable is that the drug design activity was utilized in all four years (2013–2017) the class was taught by these professors, while the case study was utilized by the experienced faculty member team. This makes drawing conclusions based on comparison of student performance between the last year of the case study and the first year of the drug design activity difficult if not impossible.

## 4. Conclusions

This article reports the design, implementation, and assessment results of an active-learning activity utilizing creative and critical thinking components. Students exhibited the ability to apply concepts learned in previous courses such as isosteric replacement, formulation, and the physical characteristics of various functional groups to new material. They also demonstrated excellent knowledge of the pharmacophoric requirements and structure activity relationships surrounding corticosteroids. The confounding variables discussed contributed notably in ascertaining assessment trends meeting the expected level of statistical significance. Despite these trends, the students and instructors explored a variety of substitution patterns reflected in the scientific literature that otherwise would not have been discussed. Continued analysis of future class performance with respect to this activity and the objectives involved are ongoing. Although the described application is for corticosteroids, similar activities could be adapted for any drug class with a well-defined structure activity relationship model such as the gonadal hormones, benzodiazepines, phenothiazines, or dihydropyridines. The authors hope the communication of our experience with this activity may encourage other faculty to design and execute similar activities that challenge students to think about medicinal chemistry in more creative and less conventional formats.

## Figures and Tables

**Table 1:** Course material covered in each of the ten segments of the endocrine course.

Segment	Material Covered
1	Hypothalamus and Pituitary Gland
2	Gonadal Hormones: Androgens
3	Gonadal Hormones: Estrogens
4	Gonadal hormones: Progestins
5	Adrenocortical Hormones: Physiology and Pharmacology
6	Adrenocortical Hormones: Medicinal Chemistry
7	Thyroid Gland
8	Bone Mineral Homeostasis
9	Pancreatic Hormones and Drugs to Treat Diabetes 1
10	Pancreatic Hormones and Drugs to Treat Diabetes 2

**Table 2:** Student Assessment Performance.

Academic Year	2013–2014	2014–2015	2015–2016	2016–2017
Students Enrolled	86	87	84	78
Performance on Objective C03	69%	82%	72%	75%
Performance on all 3 Objectives in C	76%	80%	76%	82%
Class Average on Assessment	82%	83%	86%	85%
Class Performance on Chemistry Objectives	82%	91%	86%	85%
Class Performance on Pharmacology Objectives	84%	80%	86%	85%
Percentage of Re-assessors	31%	33%	29%	30%

**Table 3:** Student Performance on Assessment Questions Mapped to Objective C03.

Question	2013–2014	2014–2015	2015–2016	2016–2017
SAR	47%	52%	73%	65%
Metabolsim	53%	87%	69%	78%
11B HSD	78%	90%	82%	74%
C9α-F	88%	94%	57%	81%
Mineralocorticoid	78%	89%	77%	79%

## Appendix A. The Learning Activity Assignment Received by Students

**PHCY 733 Endocrine**
**Day 3, PM, Activity**

In your groups, with the aid of notes, internet, etc.—answer the following questions and complete the activities.

(1) As a group, design two new drugs—one glucocorticoid and one mineralocorticoid. Be prepared to present the new drugs to the class. You can focus on whatever aspect you choose, but be sure that you can defend your design to the “FDA”! Remember that design of glucocorticoid structures are directly related to their intended use.
(2) Each group should nominate one of their members to serve on the “FDA” panel to review the proposed drugs from the other groups—they will recuse themselves from the panel to help present their group’s new drugs. Be sure to arm this person with information pertinent to the review.

## References

[1] Sanford N.S. (1965). The American College.

[2] Bonwell C.C., Eison J.A. (1991). Active Learning: Creating Excitement in the Classroom.

[3] Hernick M. (2015). Test-Enhanced Learning in an Immunology and Infectious Disease Medicinal Chemistry/Pharmacology Course. Am. J. Pharm. Educ..

[4] Mohammed A.I., Teresa A.S. (2014). Students’ Perception of an Integrated Approach of Teaching Entire Sequence of Medicinal Chemistry, Pharmacology, and Pharmacotherapeutics Courses in PharmD Curriculum. J. Pharm. Pract..

[5] Marshall L.L., Nykamp D. (2010). Active-Learning Assignments to Integrate Basic Science and Clinical Course Material. Am. J. Pharm. Educ..

[6] Kolluru S., Roesch D.M., Akhtar de la Fuente A. (2012). A Multi-Instructor, Team-Based, Active-Learning Exercise to Integrate Basic and Clinical Sciences Content. Am. J. Pharm. Educ..

[7] Brown S.D. (2010). A Process-Oriented Guided Inquiry Approach to Teaching Medicinal Chemistry. Am. J. Pharm. Educ..

[8] Alsharif N.Z., Galt K.A. (2008). Evaluation of an Instructional Model to Teach Clinically Relevant Medicinal Chemistry in a Campus and a Distance Pathway. Am. J. Pharm. Educ..

[9] Small L.D. (1964). The Identification of Organic Medicinal Compounds as Taught in Pharmaceutical Chemistry. Am. J. Pharm. Educ..

[10] Ghoneim O., Alper R.H., Szollosi D.E., Sweezy M.A., Vadlapatla R., Edafiogho I.O. (2016). Implementation of an elective course to introduce pharmaceutical sciences research. Curr. Pharm. Teach. Learn..

[11] Vahdat L. (2015). Integrating students’ learning with professional practice through laboratory and workshop based teaching in undergraduate medicinal chemistry. Pharm. Educ..

[12] Tavares M.T., Primi M.C., Silva N.A., Carvalho C.F., Cunha M.R., Parise-Filho R. (2017). Using an in Silico Approach to Teach 3D Pharmacodynamics of the Drug–Target Interaction Process Focusing on Selective COX2 Inhibition by Celecoxib. J. Chem. Educ..

[13] Satyanarayanajois S.D. (2010). Active-Learning Exercises to Teach Drug-Receptor Interactions in a Medicinal Chemistry Course. Am. J. Pharm. Educ..

[14] Kolluru S. (2012). An Active-Learning Assignment Requiring Pharmacy Students to Write Medicinal Chemistry Examination Questions. Am. J. Pharm. Educ..

[15] Roche V.F. (2009). A Receptor-Grounded Approach to Teaching Nonsteroidal Antiinflammatory Drug Chemistry and Structure-Activity Relationships. Am. J. Pharm. Educ..

[16] Tatachar A., Kominski C. (2017). Assessing a traditional case-based application exercise and a student question creation exercise on student performance and perceptions. Curr. Pharm. Teach. Learn..

[17] Shah S., Lynch L.M.J., Macias-Moriarity L.Z. (2010). Crossword Puzzles as a Tool to Enhance Learning About Anti-Ulcer Agents. Am. J. Pharm. Educ..

[18] DellaVecchia M.J., Claudio A.M., Fairclough J.L. (2017). A pharmacy student's role as a teaching assistant in an undergraduate medicinal chemistry course—Implementation, evaluation, and unexpected opportunities for educational outreach. Curr. Pharm. Teach. Learn..

[19] Josef F., Thomas G.H. (1962). 16-Ethers of 16,17-Dihydroxy Steroids of the Pregnane Series. U.S. Patent.

[20] Nomine G., Bertin D. (1964). 16,16-Dimethylcortisone Acetate,. French Patent.

[21] Beard C., Cross A.C. (1969). 11-Substituted 16α,17α-DifluoromethylenePregn-4-ene Derivatives Useful as Antiinflammatory Drugs. U.S. Patent.

[22] Beard C., Cross A.C. (1968). 16alpha,17alpha-(Dihalomethylene)pregn-4-eno[3,2-c]pyrazoles. U.S. Patent.

[23] Rosenkranz G., Velasco M. (1965). 16B-Methyl-16α,17α-Dihydroxypregnanes. U.S. Patent.

[24] Ringold H.J., Zderic J.A., Djerassi C., Bowers A. (1962). Cyclic Acetals and Ketals of the 4-Pregnene Series. German Patent.

[25] Heller M., Bernstein S. (1967). Synthesis of 16. beta.-aminopregn-5-ene-3.beta.,20.beta.-diol and related compounds. J. Org. Chem..

[26] Nathansohn G., Winters G. (1970). Antiinflammatory Steroidoxazolidines and Steroidoxazolidinooxazines. German Patent.

[27] Wechter W.J. (1967). 6-Methyl B-Norsteroids. U.S. Patent.

[28] Julia S., Neuville C., Simon P. (1962). New Derivatives of 7,7-Dimethylpregnane. Bull. Soc. Chim. Fr..

[29] Julia S., Julia M., Davis M. (1960). 7,7-Dimethyl- and 6,7,7-Trimethylcholesterol. Bull. Soc. Chim. Fr..

[30] Julia S., Julia M., Davis M. (1959). 7,7-Dimethylcholesterol. Comptes Rendus Chimie.

[31] Vermeulen C., Gijs L., Collin S. (2005). Sensorial Contribution and Formation Pathways of Thiols in Foods: A Review. Food Rev. Int..

[32] Hanze A.R., Hogg J.A., Nathan A.H. (1960). 11-Oxygenated 16α-Hydroxy-4-Amdrostene-3,17-Diones. U.S. Patent.

